# The impact of monetary incentives on referrals by traditional birth attendants for postnatal care in Nigeria

**DOI:** 10.1186/s12884-019-2313-8

**Published:** 2019-05-20

**Authors:** Adanna Chukwuma, Chinyere Mbachu, Margaret McConnell, Thomas J. Bossert, Jessica Cohen

**Affiliations:** 1 Health, Nutrition, and Population Global Practice, World Bank Group, Washington, DC 20433 USA; 2 Health Policy Research Group, College of Medicine, University of Nigeria, Enugu, Nigeria; 3 Department of Global Health and Population, Harvard T. H. Chan School of Public Health, 677 Huntington Avenue, Boston, MA 02115 USA

**Keywords:** Postnatal, Traditional birth attendants, Referrals, Incentives, Maternal, Neonatal, Health

## Abstract

**Background:**

Gaps in postnatal care use represent missed opportunities to prevent maternal and neonatal death in sub-Saharan Africa. As one in every three non-facility deliveries in Nigeria is assisted by a traditional birth attendant (TBA), and the TBA’s advice is often adhered to by their clients, engaging TBAs in advocacy among their clients may increase maternal and neonatal postnatal care use. This study estimates the impact of monetary incentives for maternal referrals by TBAs on early maternal and neonatal postnatal care use (within 48 h of delivery) in Nigeria.

**Methods:**

We conducted a non-blinded, individually-randomized, controlled study of 207 TBAs in Ebonyi State, Nigeria between August and December 2016. TBAs were randomly assigned with a 50–50 probability to receive $2.00 for every maternal client that attended postnatal care within 48 h of delivery (treatment group) or to receive no monetary incentive (control group). We compared the probabilities of maternal and neonatal postnatal care use within 48 h of delivery in treatment and control groups in an intention-to-treat analysis. We also ascertained if the care received by mothers and newborns during these visits followed World Health Organization guidelines.

**Results:**

Overall, 207 TBAs participated in this study: 103 in the treatment group and 104 in the control group. The intervention increased the proportion of maternal clients of TBAs that reported attending postnatal care within 48 h of delivery by 15.4 percentage points [95% confidence interval (CI): 7.9–22.9]. The proportion of neonatal clients of TBAs that reportedly attended postnatal care within 48 h of delivery also increased by 12.6 percentage points [95% CI: 5.9–19.3]. However, providers often did not address the issues that may have led to maternal and newborn postnatal complications during these visits.

**Conclusions:**

We show that motivating TBAs using monetary incentives for maternal postnatal care use can increase skilled care use after delivery among their maternal and neonatal clients, who have a higher risk of mortality because of their exposure to unskilled birth attendance. However, improving the quality of care is key to ensuring maternal and neonatal health gains from postnatal care attendance.

**Trial registration:**

The trial was retrospectively registered in clinicaltrials.gov (NCT02936869) on October 18, 2016.

**Electronic supplementary material:**

The online version of this article (10.1186/s12884-019-2313-8) contains supplementary material, which is available to authorized users.

## Background

At 546 per 100,000 live births, sub-Saharan Africa has the highest regional maternal mortality ratio in the world [1]. Child mortality is also highest in sub-Saharan Africa, where one child in 12 dies before their fifth birthday, 45% of whom are neonates [2]. Over 50% of maternal and newborn deaths occur in the early postnatal period, that is the first 48 h after delivery when coverage of skilled care is also at its lowest [3]. The World Health Organization recommends postnatal care for both the mother and newborn within 24 h of birth regardless of where delivery occurs [4]. Early postnatal care visits among mothers and newborns provide opportunities to check for danger signs such as bleeding and fever, facilitating early diagnosis and appropriate management of complications [see Additional file 1]. However, only about half of mothers and newborns receive postnatal care within 48 h of delivery in sub-Saharan Africa, with significant variations across countries [5]. These gaps in coverage of skilled early postnatal care represent missed opportunities to prevent maternal and neonatal death.

In Nigeria, there is a wide disparity in early postnatal care use by the location of delivery. For example, while 8 in 10 mothers who deliver in facilities receive skilled postnatal care within two days of birth, less than 2 in 10 mothers who give birth outside the facility receive skilled postnatal care [6]. Similarly, following a facility delivery, a newborn is four times more likely to receive postnatal care within two days of birth, than if delivery occurred elsewhere [6]. As a traditional birth attendant assists one in every three non-facility deliveries in Nigeria (TBA), and the TBA’s advice is often adhered to by their clients, engaging TBAs in advocacy for early postnatal care use may increase skilled care use by mothers and newborns following non-facility deliveries. A TBA is a non-formally trained and community-based provider of pregnancy-related care [7]. TBAs compete with skilled health workers for clients, primarily antenatally and at childbirth, and there is a reputational risk and the potential for client loss in future pregnancies if the TBA refers to skilled health workers [8]. Nonetheless, qualitative research indicates that even when Nigerian mothers do not receive postnatal care themselves, they often recognize the need for their newborns to receive early postnatal care, particularly for recommended immunizations [8]. Thus, recent programs that have offered TBAs monetary incentives have focused on referrals of mothers specifically to skilled providers, with mixed results, as described below.

Qualitative evaluations in Somaliland and Nigeria suggest that monetary rewards to TBAs for referrals to skilled providers predict increases in the number of women per year that received care in health facilities [9, 10]. However, neither evaluation included comparison facilities or any other counterfactual, so that increases in facility visits cannot be attributed to monetary rewards. In a recent randomized controlled trial in Kenya, TBAs were offered USD 1.20 for each antenatal care (ANC) visit made by their clients [11]. However, after adjusting for baseline covariates, the treatment effect was indistinguishable from zero at the 5 and 10% level. There is no experimental evidence of the impact of monetary incentives given to TBAs on maternal or neonatal postnatal care use. Therefore, our study uses data collected from a field experiment in Nigeria to estimate the impact of monetary rewards for maternal referrals on maternal and neonatal postnatal care use within 48 h of delivery.

## Methods

### Sample recruitment

Between August and December of 2016, the study was conducted in Ebonyi State, Nigeria where 1 in 2 mothers and 8 in 10 newborns do not receive postnatal care within the first two days of childbirth. In Ebonyi State, 4 in 10 non-facility deliveries are assisted by a TBA [6]. Using the national facility census list and via consultations with officials in the State Ministry of Health, the study team purposively selected 128 wards in Ebonyi State that had at least one primary health care facility with a maternal health care provider offering maternal postnatal care. In August 2016, 23 trained enumerators visited each ward and met with the heads of facilities to inform them of the study with a letter of introduction from the State Ministry of Health. The research team anticipated that health care providers would have received training in postnatal care. To ensure that there was minimal variation in understanding of the timing and content of postnatal care, the script for visits by enumerators to facilities included information drawn from The World Health Organization standard guidelines (see Additional file 1). In all the wards, the enumerators reminded heads of facilities about the national guidelines on postnatal care attendance for mothers and their newborns, including the need for multiple early postnatal visits within the first 24–48 h of delivery. Posters containing this information were then placed in every consulting room by the enumerators and facility heads. Facility heads were encouraged to share this information with other maternal and newborn health care providers in the facility if any. The enumerators then asked the facility heads for the names, addresses, and phone numbers of community leaders and TBAs in the ward. Community leaders were visited and asked for contact information of other TBAs in the community, in addition to verifying the names of TBAs provided by facility heads. The enumerators searched for all the TBAs whose contact details the facility head and community leader had given them. To participate in the study, TBAs had to be resident in the ward, not plan to relocate before January 2017 and provide oral informed consent. The enumerators informed TBAs about the study if they met the inclusion criteria.

### Baseline data collection

After the TBA granted informed consent, the enumerator conducted an interviewer-led survey to determine the following: demographic characteristics including age, educational level, status as head of household or primary income earner, household characteristics, and other occupation; service profile including if the TBA offered postnatal care, fees charged for services provided (where payment was in kind, we asked the TBA to approximate the monetary value of the item in the local currency), average number of postnatal care referrals per month, and average number of clients per month; as well as questions on the TBAs motivation for service, relationship with health workers, perception of facility care quality, and membership in community organizations.

All TBAs were informed of the benefits of postnatal care within 48 h of delivery for maternal and neonatal survival and encouraged to refer their maternal clients within this time window. The enumerator told the TBA that the study team would offer NGN 200 (USD 0.70) during the follow-up visit in 2–3 weeks for every maternal client the TBA provided services to if the study team was informed. This information was repeated at each visit to the TBA throughout the study. To discourage falsifying postnatal care attendance, TBAs were informed that the study team would randomly choose clients who reportedly attended postnatal care and check the registers in facilities to ascertain visits by clients. The enumerator then gave each TBA a flyer with this information on early postnatal care and the contact details of a member of the study team to call if they had concerns.

### Randomization and intervention

In September 2016, during the second visit to TBAs, enumerators asked TBAs about clients they had provided service to since the study baseline. These clients were not considered in examining treatment effects as the intervention had not commenced. Each TBA was then randomly assigned to the treatment (103 TBAs) or control group (104 TBAs) in STATA 14.2 with 50–50 probability as follows. In a dataset with all TBAs, a random uniform variable was generated, the dataset was sorted by the uniform variable, and all realizations below 0.5 randomized to the treatment group [12]. Enumerators surveyed TBAs in one group only for the rest of the study – either treatment TBAs or control TBAs and never both types – to avoid a spillover of the intervention via enumerators telling TBAs about other arms of the study. TBAs assigned to the treatment group were then informed that the study team would also offer them NGN 600 (about USD 2) for every maternal client we located who had attended maternal postnatal care within 48 h of delivery. TBAs were encouraged to refer all maternal clients, not just those with suspected complications following delivery. Treatment TBAs were also given a flyer with information on the intervention and contact details of a member of the study team to call if they had complaints or concerns. The amount of the incentive was chosen based on the Nigerian qualitative study that offered monetary incentives for facility referral and reportedly had high take-up by TBAs [10]. The magnitude relative to average earnings among TBAs is discussed in the Results section. No reward was offered for neonatal postnatal care attendance.

### Endline data collection

Endline data collection for the intervention occurred from October to November 2016. Enumerators visited all the clients whose contact details they had obtained from TBAs and surveyed them when they were at least three days post-delivery. The client survey asked if the mother had received care from the TBA, her expected and actual date of delivery, if she attended postnatal care, and if her neonate attended postnatal care. If client reported maternal or neonatal postnatal care use, the survey asked for her motivations for attending postnatal care, the date of attendance, the facility name and location, and the content of care offered her and her newborn. If clients were less than three days post-delivery, the enumerator returned to survey the client later.

### Sample size calculation and statistical analysis

The study was powered to detect a difference in the proportion of TBA clients who attend early postnatal care of 0.2 or 20 percentage points between study arms at the two-sided 5% level of significance with 90% statistical power. All analysis was conducted in STATA® 14.2 software. The unit of analysis in this study is the TBA (not the ward or client). We estimated the intention-to-treat effect of incentives for maternal early postnatal care referrals on the proportion and number of TBA maternal and neonatal clients, from the intervention rollout to the study endline that reported attending postnatal care within 48 h of delivery [13].

### Ethics approvals and consent to participate

Approval for this study was obtained from the Institutional Review Board of the Harvard T. H. Chan School of Public Health (IRB16–0923) and by the Research Ethics Committee of the State Ministry of Health, Ebonyi State (MOH/EP/021/16). All participants provided recorded oral informed consent to participate in the study.

## Results

**Fig. 1:**
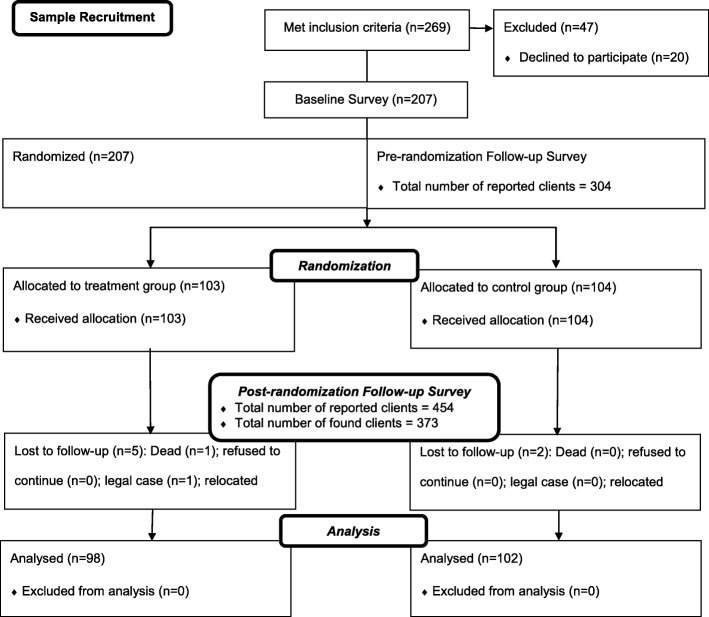
CONSORT diagram describing the flow of participants through the intervention

Out of the 269 TBAs approached by the enumerators, 47 could not be recruited for the study, either because they refused to participate or were not at the address the enumerators were given (Fig. 1). The study team recruited 207 TBAs (77%) for the study, of whom 103 were randomized to the treatment group and 104 to the control group. The control group (51.4 years) was on average seven years older than the treatment group (44.7 years) and 9.8 percentage points less likely to have any primary education (21.4 versus 11.5%). The other differences in observed pre-treatment TBA characteristics were statistically insignificant at the 5 and 10% level (Table 1). The average income per pregnancy in the treatment group was NGN 2265 (about USD 8) so that the monetary incentive offered per pregnancy of NGN 600 (about USD 2) was 25% of the average income per pregnancy. During the study, a total of 7 TBAs were lost to follow-up due to death, migration to another state, an ongoing legal dispute, and wrongful classification as TBAs. The control arm lost 2 TBAs while the treatment arm lost 5 TBAs. Thus the retention rates in the control and treatment arm were 98% and 95% respectively, and the difference of 3 percentage points [95% CI: − 2.0 – 7.9] was not statistically significant at the 5 and 10% levels.

**Table 1:** Comparison of baseline characteristics among treatment and control TBAs

Characteristic	Control TBAs [95% CI]	Treatment TBAs [95% CI]	Difference [95% CI]	*P*-value
	(*n* = 104)	(*n* = 103)		
Age in years	51.423 [47.597–55.249]	44.757 [41.585–47.930]	6.666 [1.721–11.611]	0.009
Female gender	0.971 [0.938–1.004]	0.942 [0.896–0.988]	0.029 [− 0.027–0.085]	0.302
No formal education	0.462 [0.364–0.559]	0.388 [0.293–0.484]	0.073 [− 0.063–0.209]	0.289
Some or completed primary education	0.115 [0.053–0.178]	0.214 [0.133–0.294]	− 0.098 [− 0.199–0.003]	0.057
Some or completed secondary education	0.337 [0.244–0.429]	0.311 [0.220–0.402]	0.026 [−0.103–0.155]	0.693
Some or completed post-secondary education	0.087 [0.032–0.141]	0.087 [0.032–0.143]	−0.001 [− 0.078–0.077]	0.983
Average income per pregnancy (Naira)	1790.962 [1430.692–2151.231]	2265.437 [1680.668–2850.206]	−474.475 [− 1155.743–206.793]	0.171
Offers maternal or neonatal postnatal care	0.683 [0.592–0.774]	0.592 [0.496–0.689]	0.090 [−0.041–0.222]	0.178
Monthly average number of delivery clients	13.808 [−5.194–32.809]	9.553 [−0.005–19.112]	4.254 [−16.952–25.460]	0.693
Average number of maternal clients who attended PNC	0.212 [0.002–0.421]	0.175 [0.021–0.328]	0.037 [−0.222–0.295]	0.779
Average number of neonatal clients who attended PNC	0.202 [−0.009–0.412]	0.194 [0.060–0.328]	0.008 [− 0.241–0.257]	0.927
Literate in English	0.433 [0.336–0.530]	0.456 [0.358–0.554]	−0.024 [− 0.160–0.113]	0.734
Literate in other language	0.260 [0.174–0.345]	0.282 [0.193–0.370]	− 0.022 [− 0.144–0.100]	0.724
Membership of any association	0.846 [0.776–0.917]	0.864 [0.797–0.931]	−0.018 [− 0.115–0.079]	0.716
Main income earner in household	0.250 [0.165–0.335]	0.272 [0.184–0.359]	− 0.022 [− 0.143–0.099]	0.722

Note - *CI* confidence interval, *PNC* postnatal care, *TBA* traditional birth attendant

Between randomization in September 2016 and the study endline in November 2016, TBAs in the control group reported 2.1 clients on average, while TBAs in the treatment group reported 2.4 clients on average. The difference of − 0.35 clients [95% CI: − 1.4 – 0.7] between groups was not statistically significant at the 5 and 10% levels. Of the 454 clients that TBAs reported providing services to between the randomization and the study endline, the study team found a total of 373 clients (82%), of which 21 did not grant informed consent to continue with the survey. Clients that could not be found had moved out of the TBA’s community and had no contact phone number. The proportion of cases where the number of clients reported by TBAs equaled the number of clients found by the study team was 81.7% in the control group and 89.3% in the treatment group. The difference of 7.6 percentage points was not statistically significant at the 5 and 10% levels [95% CI: -0.02 – 0.17]. Out of the 352 clients surveyed, 341 were delivered outside a health facility, while 11 clients were delivered in health facilities by TBAs. Of the 109 mothers that reported attending postnatal care from the study baseline to endline (41 between the study baseline and intervention roll-out and 68 between intervention roll-out and the study endline), the study team randomly sampled 17 cases (16% of 109) to ascertain attendance in the health facility. The health facilities had postnatal care registers in 13 cases (76% of 17). In cases where there were registers, the client’s name under the appropriate date was found in 7 cases (54% of 13). In the 6 cases where the client’s name was not found despite the presence of a postnatal care register, the register had no dates, indicating that record-keeping may not have been accurate. The study team rewarded treatment TBAs based on reported early postnatal care attendance by their clients.

In Table 2, we show that the probability that a maternal client of a treatment TBA attended postnatal care within 48 h of delivery was 15.4 percentage points [95% CI: 7.9–22.9] higher than for maternal clients of control TBAs. The confidence intervals for the probability that a maternal or neonatal client of a control TBA attended postnatal care within 48 h contains the null value, so we cannot reject the hypothesis that the effect in control TBAs is zero. While the intervention rewarded treatment TBAs solely for maternal clients that attended postnatal care within 48 h of delivery, the probability that a neonatal client of a treatment TBA attended postnatal care within 48 h of delivery in the treatment group was 12.6 percentage points [95% CI: 5.9–19.3] higher than for neonatal clients of control TBAs. These findings were robust to re-specification adjusting for baseline characteristics of the TBAs. Among TBAs that did not report having any clients or referrals, the probability that their neonatal or maternal client attended postnatal care within 48 h of delivery is recorded as zero (rather than infinity to accommodate an intention-to-treat analysis). As this conflates TBAs who had no clients over the intervention duration with TBAs who had clients all of which did not attend early postnatal care, we re-estimated the effect of the intervention on the number of maternal and neonatal clients that attended postnatal care within 48 h of the intervention. As stated above, the number of maternal clients reported by TBAs was similar in both groups, so that the effect of the intervention on the number of clients who attend early postnatal care is independent of the number of clients the TBA provided service to over the intervention duration. As shown in Table 3, treatment TBAs referred 0.31 more maternal clients [95% CI: 0.1–0.5] and 0.26 more neonatal clients [95% CI: 0.1–0.5] than control TBAs on average. These findings are also robust to re-specification adjusting for baseline characteristics of the TBAs.

**Table 2:** Intention-to-treat effects of performance-based monetary incentives to TBAs on the proportion of maternal and neonatal clients that attended postnatal care within 48 h of delivery

	Maternal clients		Neonatal clients	
	Mean [95% CI]	Mean [95% CI]	Mean [95% CI]	Mean [95% CI]
Treatment effect	0.154 [0.079–0.229]	0.149 [0.068–0.229]	0.126 [0.059–0.193]	0.123 [0.049–0.198]
*P*-value	0.0001	0.001	0.0001	0.002
Control group mean	0.044 [−0.009–0.097]	0.067 [− 0.319–0.454]	0.032 [− 0.015–0.079]	−0.041 [− 0.397–0.315]
R-squared	0.074	0.216	0.062	0.155
Observations	207	207	207	207
Control for baseline characteristics of TBA?	No	Yes	No	Yes

Note - *CI* confidence interval, *PNC* postnatal care, *TBA* traditional birth attendant

**Table 3:** Intention-to-treat effects of performance-based monetary incentives to TBAs on the number of maternal and neonatal clients that attended postnatal care within 48 h of delivery

	Maternal clients		Neonatal clients	
	Mean [95% CI]	Mean [95% CI]	Mean [95% CI]	Mean [95% CI]
Treatment effect	0.312 [0.111–0.514]	0.297 [0.079–0.516]	0.263 [0.081–0.446]	0.263 [0.066–0.460]
P-value	0.003	0.008	0.005	0.009
Control group mean	0.173 [0.031–0.315]	0.630 [−0.413–1.673]	0.135 [0.006–0.263]	0.115 [− 0.826–1.057]
R-squared	0.044	0.175	0.038	0.176
Observations	207	207	207	207
Control for baseline characteristics of TBA?	No	Yes	No	Yes

Note - *CI* confidence interval, *PNC* postnatal care, *TBA* traditional birth attendant

The intention-to-treat estimates retain all TBAs enrolled at baseline in the study within the randomized arms. We also estimated per-protocol effects, restricted to TBAs who were retained in the study. In Table 4, we show that the study findings are robust to this analysis.

**Table 4:** Per-protocol effects of performance-based monetary incentives to TBAs on the proportion of maternal and neonatal clients that attended postnatal care within 48 h of delivery

	Maternal clients		Neonatal clients	
	Mean [95% CI]	Mean [95% CI]	Mean [95% CI]	Mean [95% CI]
Treatment effect	0.163 [0.086–0.240]	0.157 [0.075–0.240]	0.133 [0.064–0.202]	0.130 [0.053–0.206]
*P*-value	0.0001	0.0001	0.0001	0.001
Control group mean	0.045 [−0.009–0.099]	0.015 [− 0.389–0.420]	0.033 [− 0.016–0.081]	−0.007 [− 0.382–0.368]
R-squared	0.081	0.233	0.068	0.166
Observations	200	200	200	200
Control for baseline characteristics of TBA?	No	Yes	No	Yes

**Fig. 2:**
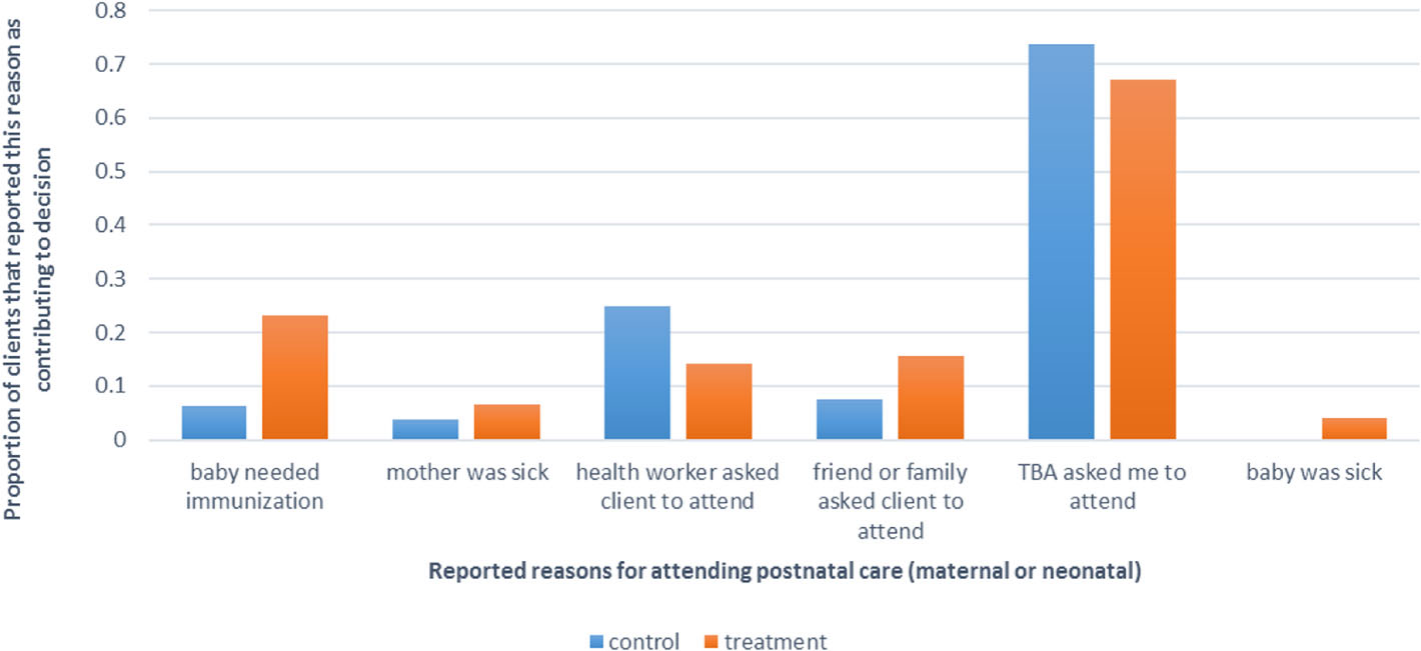
Self-reported motivations for postnatal care attendance among TBA clients

**Fig. 3:**
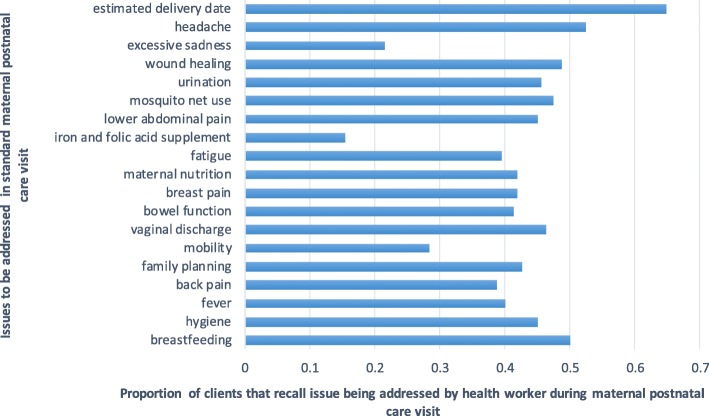
Self-reported experience of maternal postnatal care

**Fig. 4:**
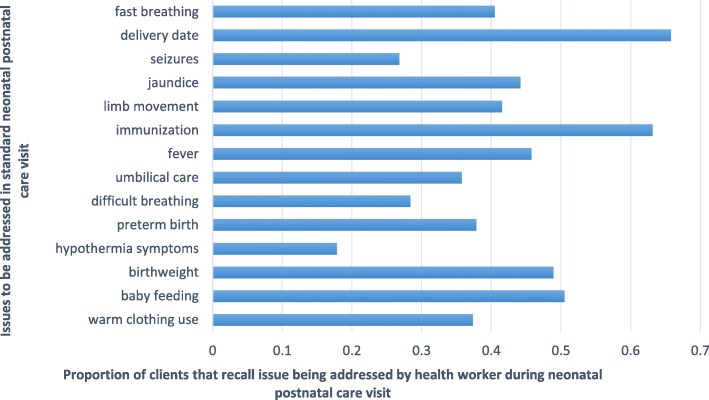
Self-reported experience of neonatal postnatal care

We then examined self-reported motivations for early postnatal care attendance, allowing for more than one motivation to be identified for the maternal and neonatal visit. We did not examine separate motivations for attending neonatal and maternal early postnatal care. In Fig. 2, we show that in 70% of cases, the reported reason for attending maternal and neonatal early postnatal care among clients of both treatment and control groups was the advice of the TBA. To a lesser extent, advice from health workers, friends, and family, also reportedly motivated early postnatal care attendance among TBA clients. Among most clients, ill-health of the mother or neonate following delivery was not the primary motivation for attending early postnatal care. This finding may, however, point to a pattern of primarily referring clients that did not have complications [8]. We thus pooled all the client data to examine the probability of attending postnatal care within 48 h of delivery among maternal and neonatal clients with or without complications. We found that attendance of early postnatal care was not predicted by the self-reported incidence of complications in both mothers and their neonates (not shown). We also asked mothers to recall if essential components of an early postnatal care consultation, as stipulated in World Health Organization guidelines (see Additional file 1), were addressed during the neonatal and maternal visit [4]. For both maternal and neonatal postnatal care visits, clients reported that an average of 42% of the recommended components was addressed by the provider (Figs. 3 and 4).

## Discussion

This paper shows that monetary rewards for maternal referrals by TBAs increased the proportion of maternal clients that attended postnatal care within 48 h of delivery by 15.4 percentage points, and the proportion of neonatal clients that attended postnatal care within 48 h of delivery by 12.6 percentage points. To our knowledge, this is the first field experiment examining the impact of performance-based monetary incentives on referrals by TBAs for maternal and neonatal postnatal care attendance in Africa. Our findings agree with evidence from qualitative evaluations of programs in Somaliland and Nigeria suggesting that monetary incentives for performance may motivate postnatal referrals of mothers to skilled providers by TBAs [9, 10].

Only 16% of mothers and 13% of newborns in the intervention group reported receiving postnatal care within the first 48 h, leaving most maternal and neonatal TBA clients in the intervention group without skilled care during this critical period. These figures are below the national prevalence of receiving maternal and neonatal postnatal care within 48 h of delivery following facility delivery of 79 and 28% respectively [6]. Even when TBAs are motivated to refer their clients for care, there may be other barriers to skilled postnatal care use that may prevent compliance with referrals, including disrespectful skilled care, low service delivery capacity, the cost of treatment, transportation difficulty, and lack of understanding among mothers about the need for skilled postnatal care in the absence of complications [8, 14, 15]. Therefore, successful efforts aimed at increasing the use of skilled maternal health care in Nigerian facilities may have to address multiple barriers to care-seeking behavior in these contexts.

This paper adds to the evidence base on the effectiveness of performance-based monetary incentives in motivating human behavior [16, 17]. Much of the research on performance-based monetary incentives in maternal and child health has focused on cash transfers to mothers to encourage the use of skilled and facility care. A 2013 review of the impact of conditional cash transfers on maternal and newborn health, included two programs in Honduras and El Salvador that rewarded mothers for postnatal visits, in addition to improvements to the quality of facility care and nutrition services, as well as health promotion [18–20]. In both cases, the interventions had no impact on postnatal care use. In Nigeria, an intervention that offered mothers NGN 1000 (USD 3.33) for the first neonatal immunization and postnatal visit with family planning advice for the mother also did not have any impact on postnatal care use [21]. A quasi-experimental evaluation of an Indonesian program gave cash transfers of up to $220 to households conditioned on four ANC visits, iron tablets during pregnancy, assisted delivery, and two postnatal visits. This program also made improvements to the quality of facility care so that program effects cannot be attributed to cash transfers only. The program reportedly increased the probability of at least two postnatal care visits by 5.1 percentage points [22]. Thus, the impact of cash transfers to mothers for postnatal care attendance is mixed, highlighting the need for replication of this field experiment on monetary incentives for TBA referrals in other settings and perhaps with varying incentive amounts.

Follow-up surveys with maternal clients of TBAs in this study that attended early postnatal care suggested that the predominant reason for attendance was the influence of the TBA. One of the most commonly reported components of early postnatal care among neonates was immunization, which is often the reason for the first routine neonatal postnatal care visit. Client reports suggested that early postnatal care visits often did not address issues that are considered critical to maternal and newborn survival, such as fever to identify infection onset and warm clothing to prevent hypothermia. Low adherence to guidelines for early postnatal care among skilled health workers has been noted in other settings [23, 24]. As the benefits of maternal and neonatal early postnatal care attendance are conditional on high-quality service delivery, these gaps in adherence highlight the critical need for policy attention to be given to improving quality of care before or as a complement to interventions aimed at increasing postnatal care use. Self-reports on visit components may have been affected by the recall of service experiences and should be treated with caution.

The primary strength of this study is the use of randomization to attempt to construct a credible counterfactual for the intervention group. Baseline comparison of the treatment and control groups indicates they were reasonably balanced on observed characteristics. However, there were statistically significant differences in average age and education. During the intervention, the difference in probability of retaining a TBA in the treatment and control group was not statistically significant at the 5 or 10% level. The difference in the proportion of cases where the number of clients reported by TBAs equaled the number of clients found by the study team was also not statistically significant at the 5 or 10% level. A high percentage (92.6%) of TBAs approached to participate in this study accepted to do so, indicating the potential for widespread take-up if this intervention were implemented to increase postnatal care use. While the rates of TBA refusal to participate were low, it may be that TBAs that refused to participate differ in significant ways from those that accepted to participate, with implications for the generalizability of our findings. This study has other limitations. We repeatedly gave information to TBAs in the control group on the benefits of early postnatal care referrals, akin to treatment TBAs, to isolate the effect of monetary incentives on early postnatal care attendance. We are thus unable to explore interactions between information on the benefits of early postnatal care attendance and monetary incentives to TBAs for early postnatal care referrals. While we provide evidence that the TBA’s advice influenced early postnatal care attendance, we are also unable to definitively explore the mechanisms intervening the offer of monetary incentives and early postnatal care attendance. Specifically, as TBAs, rather than their clients, were the units of randomization, we are unable to explore interactions of client characteristics with the intervention systematically. Another weakness of this study is the reliance on self-reports from clients on early postnatal care attendance, subject to recall bias, rather than facility records due to the poor information systems within the study context. In cases where facility records were available, postnatal care attendance was verified. However, it may be that in cases where self-reports were not verified, women reported receiving care despite not visiting the facility. In scaling up this intervention, efforts to strengthen the quality and regularity of routine facility data would be an important way of reducing the cost of implementation while improving program monitoring and evaluation overall. This study would also have benefitted from a longer follow-up which was prevented by communal clashes due to land disputes in several villages with TBAs in the study. Furthermore, as detailed cost data were not collected, outside the incentive amounts, we did not explore the cost-effectiveness of this intervention. Future research on this subject should explore the comparative cost-effectiveness of this intervention relative to other means of increasing postnatal care use, accounting for costs beyond incentive amounts including travel time for TBAs and follow-up on clinic visits. There are also outstanding research questions relating to interactions of the financial incentive treatment with TBA, facility, and client characteristics using sufficiently powered studies [25]. Comparisons of the effectiveness of monetary incentives for maternal and neonatal referrals, as well as with cash transfers to clients for maternal and neonatal care use may also be useful.

## Conclusions

This study shows that motivating TBAs using monetary incentives can increase early postnatal care use among their clients, who have a higher risk of maternal and neonatal complications because of their exposure to unskilled birth attendance. Policy aimed at improving early postnatal care uptake using performance-based monetary incentives directed at TBAs should address improvements in the quality of postnatal care provision so that increases in postnatal care demand can translate to maternal and neonatal health gains.

## Additional file

Additional file 1: 2013 WHO Recommendations on early postnatal care for mothers and newborns [4]. Table showing a description of WHO Recommendations on early postnatal care and selected variables in the analysis of self-reports of quality of care. (DOCX 18 kb)

## Abbreviations

ANC: Antenatal care
CI: Confidence interval
NGN: Nigerian Naira
PNC: Postnatal care
TBA: Traditional birth attendant
USD: United States Dollar
