# Supplementary material for: The impact of monetary incentives on referrals by traditional birth attendants for postnatal care in Nigeria
Source: BMC Pregnancy Childbirth. 2019 May 20;19:150. doi: 10.1186/s12884-019-2313-8 (PMC6526614; doi:10.1186/s12884-019-2313-8)
Supplement: Supplementary file 1 — 2013 WHO Recommendations on early postnatal care for mothers and newborns [4]. Table showing a description of WHO Recommendations on early postnatal care and selected variables in the analysis of self-reports of quality of care. (DOCX 18 kb) [file 12884_2019_2313_MOESM1_ESM.docx]

2013 WHO Recommendations on early postnatal care for mothers and newborns [4].

| **Theme** | **Recommendation** | **Selected variables in self-reports of quality of care** |
| --- | --- | --- |
| **Postnatal care for the newborn** | | |
| Assessment of the baby | The following signs should be assessed during each postnatal care contact and the newborn should be referred for further evaluation if any of the signs is present: stopped feeding well, history of convulsions, fast breathing (breathing rate ≥60 per minute), severe chest in-drawing, no spontaneous movement, fever (temperature ≥37.5 °C), low body temperature (temperature <35.5 °C), any jaundice in first 24 hours of life, or yellow palms and soles at any age. | Fast breathing, seizures, jaundice, limb movement, fever, difficult breathing, delivery date. |
| Exclusive breastfeeding | Mothers should be counselled and provided support for exclusive breastfeeding at each neonatal postnatal contact. | Delivery date and baby feeding. |
| Cord care | Daily chlorhexidine (7.1% chlorhexidine digluconate aqueous solution or gel, delivering 4% chlorhexidine) application to the umbilical cord stump during the first week of life. | Umbilical care and delivery date. |
| Other postnatal care for the newborn | Bathing should be delayed until 24 hours after birth. If this is not possible due to cultural reasons, bathing should be delayed for at least six hours; appropriate clothing of the baby for ambient temperature is recommended; the mother and baby should not be separated and should stay in the same room 24 hours a day; communication and play with the newborn should be encouraged; immunization should be promoted as per existing WHO guidelines; preterm and low-birth-weight babies should be identified immediately after birth and should be provided special care as per existing WHO guidelines. | Delivery date, immunization, preterm birth, hypothermia, birth weight, and warm clothing. |
|  |  |  |
| **Postnatal care for the mother** | | |
| Assessment of the mother: first 24 hours after birth | All postpartum women should have regular assessment of vaginal bleeding, uterine contraction, fundal height, temperature and heart rate (pulse) routinely during the first 24 hours starting from the first hour after birth; blood pressure should be measured shortly after birth; if normal, the second blood pressure measurement should be taken within six hours; urine void should be documented within six hours. | Delivery date, urination, vaginal discharge, and fever. |
| Assessment of the mother: beyond 24 hours after birth | At each subsequent postnatal contact, enquiries should continue to be made about general well-being and assessments made regarding the following: micturition and urinary incontinence, bowel function, healing of any perineal wound, headache, fatigue, back pain, perineal pain and perineal hygiene, breast pain, uterine tenderness and lochia; breastfeeding progress should be assessed at each postnatal contact; at each postnatal contact, women should be asked about their emotional wellbeing, what family and social support they have and their usual coping strategies for dealing with day-to-day matters. All women and their families/partners should be encouraged to tell their health care professional about any changes in mood, emotional state and behavior that are outside of the woman’s normal pattern. | Delivery date, excessive sadness, wound healing, urination, lower abdominal pain, breastfeeding, breast pain, bowel function, vaginal discharge, back pain, and hygiene. |
| Counselling | All women should be given information about the physiological process of recovery after birth, and that some health problems are common, with advice to report any health concerns to a health care professional, in particular: signs and symptoms of PPH: sudden and profuse blood loss or persistent increased blood loss, faintness, dizziness, palpitations/tachycardia; signs and symptoms of pre-eclampsia/eclampsia: headaches accompanied by one or more of the symptoms of visual disturbances, nausea, vomiting, epigastric or hypochondrial pain, feeling faint, convulsions (in the first few days after birth); signs and symptoms of infection: fever, shivering, abdominal pain and/or offensive vaginal loss; signs and symptoms of thromboembolism: unilateral calf pain, redness or swelling of calves, shortness of breath or chest pain; nutrition; hygiene, especially handwashing; birth spacing and family planning; safer sex including use of condoms; insecticide-impregnated bed nets; mobilizing as soon as appropriate. | Headache, mosquito net use, lower abdominal pain, fatigue, feeding (maternal), mobility, and family planning. |
| Iron and folic acid supplementation | Iron and folic acid supplementation should be provided for at least three months. | Delivery date and iron and folic acid supplement. |
| Prophylactic antibiotics | The use of antibiotics among women with a vaginal delivery and a third or fourth degree perineal tear. | Wound healing. |
